# High altitude balloon testing of Arduino and environmental sensors for CubeSat prototype

**DOI:** 10.1016/j.ohx.2022.e00329

**Published:** 2022-06-16

**Authors:** Kenjiro S. Lay, Lingqi Li, Masataka Okutsu

**Affiliations:** a Penn State University, 201 Old Main, University Park, PA 16802, USA; b Penn State Abington, 1600 Woodland Rd, Abington, PA 19001, USA

**Keywords:** CubeSat, Arduino, APRS, Weather balloon, High altitude testing

## Abstract

CubeSats were conceived with an aim to provide students with hands-on, design, build, and test experiences on spacecraft. Many education-class CubeSats keep the cost of the projects low with the use of commercial off-the-shelf (COTS) products. But using parts not designed for space missions often means a compromise in performance (e.g., low sensor accuracy, low power efficiency) and reliability, which makes component testing a necessary part of the development process. Unfortunately, there is no single lab equipment that can test the integrated features of CubeSats, including the radio communication over ranges of altitudes and distances. It has been pointed out that a high altitude reached by a weather balloon offers an environment similar to the space environment. This paper describes a balloon flight testing of Arduino and sensors for a CubeSat “prototype”—a preliminary mock-up model used for hardware selection and validation during the initial building phase. Atmospheric pressures and temperatures were measured throughout the balloon flight. The measured pressures were validated by comparing Arduino’s pressure altitudes against the GPS altitudes, and the measured temperatures were assessed against the standard atmosphere model.

## Highlights

• A flight experiment on Arduino and sensors was conducted using a weather balloon.
• The high altitudes offer conditions that are similar to those of space.
• Thermal and barometric data were collected during the flight.
• The calculated pressure altitudes were validated against the GPS altitudes.

## Graphical abstract

**Figure:**
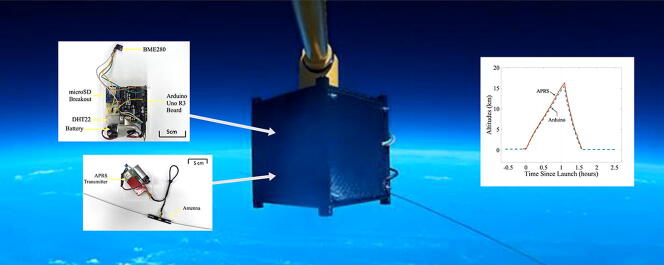

**Specifications table:** 

Hardware Name	Arduino-Based CubeSat Prototype
Subject Area	Educational tools and open source alternative to existing infrastructure
Hardware Type	Field measurements and sensors
Closest Commercial Analog	No commercial analog is available
Open Source License	GNU GPL 3.0
Cost of Hardware	US ∼$374
Source File Repository	https://doi.org/10.17605/OSF.IO/7XR38

## 1 Hardware in context

### 1.1 Arduino-based CubeSats

The dimensional standard of CubeSats, with their volumes constrained to a few liters, was conceived with an aim to get students involved in space sciences and technologies. In addition to their small sizes, CubeSats can keep the cost of development low by relying on commercial off-the-shelf (COTS) products. An open source approach to CubeSats has added benefits in that the users can share and modify resources [1]. The advantages of free, open source hardware for scientific research equipment are discussed in detail by Pearce [2], [3].

One open source COTS product that has a potential application for student- or amateur-built CubeSats is Arduino [4], a popular microcontroller programmable in C/C++ via USB port. Arduino has been used in “prototypes” of CubeSats to demonstrate such functions as weather forecasting [5], attitude determination [6], and demonstration of preliminary thermal testing [7]. We note that in this paper the term “prototype” is used broadly, referring to a range of preliminary models that are partially built and at various levels of fidelity.

As for Arduino-based CubeSats that have been actually deployed into orbit, a notable example is ArduSat [8], [9], [10], referring to the three 1-U CubeSats that were launched in 2013 and 2014. These ArduSats ran on custom-built Arduino and housed a camera and sensor suite capable of measuring acceleration, orientation, rotation, magnetic forces, pressure, temperature, and luminosity [11]. The details of science conducted by ArduSats (or by any other Arduino-based CubeSats) have not appeared in peer-reviewed publications.

Given that Arduino microcontrollers are beginner friendly yet capable for advanced projects, Arduino is a good starting point for CubeSats built by student teams. In the context of education-class CubeSats, there is a significant advantage in Arduino being popular and open source, as there are a large quantity of resources (e.g., examples, tutorials) available on the Internet. (Raspberry Pi is comparable to Arduino in terms of the cost, open source accessibility, ease of development, and technical maturity. The thermal and environmental sensors used in this study can work for both Arduino and Raspberry Pi.).

The COTS products keep the cost of CubeSats low, but often suffer in performance and reliability as the parts used are not developed for the intended space mission. As such, verification tests that can be performed easily and inexpensively are a necessary part of the development cycle. Unfortunately, no single lab equipment could test integrated features of CubeSats, including the radio communication over ranges of altitudes and distances.

### 1.2 APRS for CubeSats

Our CubeSat-inspired payload made use of the Automatic Packet Reporting System (APRS) to transmit its GPS coordinates during the balloon flight. Although using GPS in space would require special licensing, APRS as a method to send mission data has potential application to education-class CubeSats. This section provides a brief overview of APRS in this context.

Amateur radio has been used for communications in orbiting satellites since 1961 [12]. Radio communication requires an agreed-upon method to encode and decode information, and for balloons and satellites, the current de facto standard is the AX.25 protocol [13]. When the spacecraft transmits the mission data over the radio, it is useful to also attach other information (e.g., timestamp and identifiers) to the data. Those grouped data is referred to as a data packet. One well-known application of the AX.25 protocol is the APRS, which is widely used for tracking balloons [13], [14]. Being developed by Bob Bruninga in 1984, the two-way, real-time digital communication of APRS enables all devices in the local area to share the data, which are in turn made accessible via the Internet [15]. The APRS radio frequencies depend on the regions, e.g., 144.39 MHz for North America, 144.64 MHz for China, and 144.80 MHz for Europe (including all of Russia) and many African countries. The standard for space appears to be 145.825 MHz; this frequency has been used for the APRS communication by the International Space Station (ISS), PCSat-1 and 2, and PSAT.

In 2001, PCSat-1 and Sapphire (which were launched by the same rocket) became the first spacecraft to communicate with the ground using APRS. During one year of operation, the PCSat-1 (short for Prototype Communication Satellite) used APRS to communicate with more than two thousand users [16]. Sapphire used the micromachined infrared detectors and allowed the public to use the spacecraft instruments for photography [17]. For university-built satellites, the PCSat-1 and Sapphire Satellite were relatively large, weighing 10 kg and 20 kg, respectively. By 2008, however, APRS was already being considered for CubeSats, much smaller categories of spacecraft [18]. In 2015, the U.S. Naval Academy’s PSAT (short for Parkinson Satellite) became the first CubeSat to employ APRS. During the first 18 months of orbital operation, PSAT sent telemetry and mission data to the network of users worldwide [19].

For education-class CubeSats, the fact that APRS has a built-in communication network worldwide is a noteworthy advantage. Generally, an orbiting satellite can communicate with a ground station only when the vehicle is flying over the station. The spacecraft in low Earth orbit (LEO) and the ground station are in the line of sight typically less than ten times a day with each pass lasting fewer than ten minutes (with some passes being only a few minutes long). The limited opportunity and duration for acquisition of signals (AOS) pose operational challenges for inexperienced teams. CubeSats that communicate using APRS, on the other hand, have a “global coverage,” because there is an existing network of APRS users worldwide. The received APRS data are instantly shared over the Internet, so a mission operator (who can be located anywhere in the world) can retrieve the data in nearly real time. Because education-class CubeSats are typically not concerned about classified information, being able to rely on an existing network of users is an advantage.

### 1.3 Background on high altitude testing for student-built prototypes of small satellites

Although there is no special line that separates the sky from space, one convention used internationally for “edge of space” is 100 km. Latex weather balloons fly to an altitude of 15–30 km, which is closer in distance to the sea level than the typical orbital altitudes of CubeSats (e.g., 200–1,000 km). However, due to the exponential nature of the atmospheric decay, the atmospheric pressures at altitudes of 15 km (nearly at the upper edge of the troposphere) and 30 km (in the stratosphere) are already 12% and 2% of the sea level values, respectively. Likewise, the densities at those altitudes are 16% and 1.5% of the sea level values, respectively. In many ways, therefore, the flight environments for balloons are actually closer to space than to the ground.

The atmospheric temperatures at altitudes of 15 km and 30 km are −57 °C and −47 °C, respectively. Exposure to low temperature is a serious consideration, as the lower bound of many electronic components are around −40 °C. But the low atmospheric density also means that the effects of conduction and convection by the ambient air are diminishingly small. Thus, despite the ambient air being very cold, it is possible for the surface of the payload facing the sun to become hotter than it would be near the ground [20]. Electronic components in an enclosed structure must anticipate both the cold and the hot temperatures. Unfortunately, it is difficult to predict the internal temperature of a payload flown on a balloon, being subject to the range of altitudes (i.e., varying atmospheric temperatures, pressures, and densities). Even the surface materials and color of the paint will affect its temperature, which is also different from any given location within the enclosed structure. Experiences from balloon flights show that a typical payload would need to be prepared for a thermal range of −50 °C to 50 °C [21] which is comparable to that for CubeSats flown in low Earth orbits, e.g., −40 °C to 70 °C [22].

Given the similarity in their flight environments, balloon flights have been used for preliminary validation of instruments intended for spaceflights. A number of such projects have been carried out with the support from the Air Force, the National Science Foundation (NSF), and the National Aeronautics and Space Administration (NASA) [23]. If a few hours of flight duration is sufficient, a small latex weather balloon (i.e., sounding balloon) offers the advantage of a rapid flight cycle [14]. Weather balloons were successfully used to validate the long-range radio operated in harsh space-like environments [24], [25] and to test the active illumination method for tracking spacecraft’s position and altitudes [26].

A substantially longer flight is possible with the use of larger research balloons. A senior design team from the New Mexico State University, in collaboration with NASA’s Columbia Scientific Balloon Facility, conducted a balloon experiment on a prototype of a small satellite; the team reported that the flight test helped them identify weaknesses in their antenna system and verify such features as remote control systems that would be needed for spaceflights [27]. As for opportunities for student teams, one notable program (in the U.S.) is the High Altitude Student Platform (HASP), collaboratively supported by the NASA Balloon Program Office (BPO) and the Louisiana Space Consortium (LaSPACE). The HASP vehicle is designed to carry eight small payloads and four large payloads using a small zero pressure polyethylene film balloon with a flight duration of 15–20 h [21]. Those payloads are launched to an altitude of ∼36 km yearly from the NASA balloon launch facility at Fort Sumner, New Mexico [21].

### 1.4 Thermal aspects of balloon tests of CubeSats

As discussed in the previous section, operational thermal ranges of typical CubeSat missions are −40 °C to 70 °C, which is comparable to −50 °C to 50 °C of typical high altitude balloon. Those operational ranges have significant overlap to those of many electronic components (e.g., −40 °C to 85 °C, often narrower). As such, building a CubeSat using inexpensive COTS products would mean that validation tests are necessary to confirm if it could operate over the required thermal range.

Of course, many of the questions related to the reliability at the thermal bounds can be bypassed by purchasing more capable (but often more costly) equipment. Such a solution is beyond the scope of present discussion which concerns an open source, inexpensive COTS-product approach for education-class CubeSats.

The thermal ranges reported by the manufacturers often correspond to those for sustained operation under the standard atmospheric conditions. In balloon flights, the atmospheric properties change as a function of altitudes, and many of the electronic components are housed inside of an enclosed structure that provides partial thermal insulation. In actual CubeSat missions (outside of the atmosphere), a CubeSat placed in a low Earth orbit undergoes a thermal cycle: within a period of approximately 90 min, the spacecraft would repeatedly fly in the sun-lit side and the shadow side of the Earth. Given the dynamic nature of the operational thermal environments, selecting COTS products for education-class CubeSats would not be as straightforward as tasks as simply reading the thermal operational range noted in the product information.

**Fig. 1:**
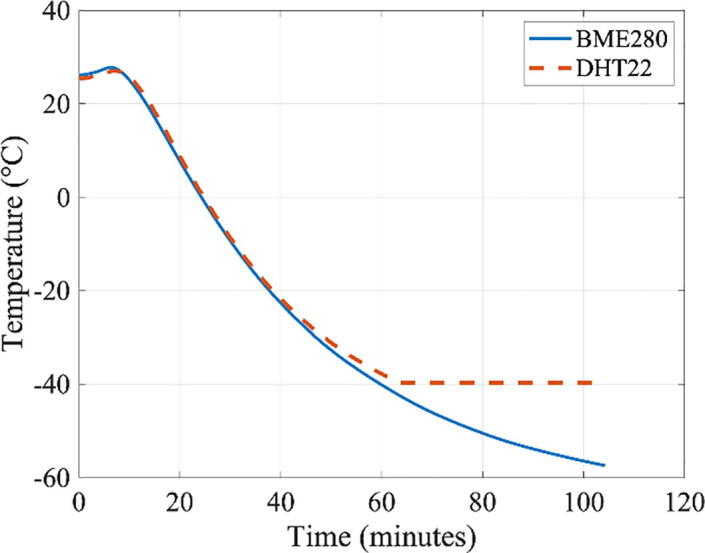
The temperature inside of the CubeSat prototype body in a deep freezer at −70 °C (∼1 atm).

To illustrate the nature of the problem, we describe a sample experiment in which a Arduino in an enclosed structure, conforming to the dimensional standard of CubeSat, was subjected to a temperature of −70 °C (using a deep freezer) for a prolonged duration. In this experiment, temperature inside an enclosed box was measured using the DHT22 and BME280 sensors, which were connected to an Arduino Uno board. The DHT22 and BME280 sensors, the Arduino Uno board, and the battery all had the lower bounds of their operational range as −40 °C, according to the respective manufacturers. Fig. 1 shows the temperature inside of the enclosed CubeSat body being measured by the DHT22 and BME280 sensors. Although the freezer temperature was −70 °C, the air temperature inside the enclosed box remained much warmer for some time, because the freezer must first cool the box’s structure, which in turn cools the air inside the box. Greater thermal insulation on the outer surface would reduce the rate of cooling (i.e., the slope of the temperature–time relationship would be shallower). The small difference in the temperature readings between the DHT22 and BME280 sensors can be explained by the fact that these sensors have ±0.5 °C and ±1.0 °C accuracies, respectively, and that they are placed in different locations within the box. The internal temperature reached the operational limit of −40 °C after a little over an hour, when the value reported by DHT22 “froze” also at −40 °C. But the BME280 sensor and Arduino continued to operate further—up to 1 h 45 min into the experiment, when Arduino finally stopped working, likely due to the temperature of the 9 V Energizer Ultimate Lithium Battery dropping below its operational lower limit of −40 °C. (After the experiment, Arduino and the battery became functional again.) An experiment like this informs that some thermal insulations would be required for the battery, and that BME280 has a higher tolerance for cold temperature than the DHT22—even though the reported lower bounds of the operational range were −40 °C for both sensors (as well as for Arduino). More discussions on preliminary thermal validation tests can be found in Ref. 7.

Thermal validation testing is necessary to confirm the operability and survivability of electronics components, especially since the thermal ranges of many COTS electronic components are comparable to the required operational range for space missions. Testing is necessary also because (in a thermally dynamic environment) the temperature of a given electronic component is different from the temperature of the outer surface or of the surrounding.

### 1.5 Limitations of balloon experiments

Although a flight environment of a balloon payload is similar to that of an orbiting CubeSat, a balloon test cannot simulate all environmental conditions that a CubeSat would undergo during its life cycle. Vibration is one such example. In an actual space mission, a CubeSat would be launched by a rocket, a rough ride that can sometimes damage the spacecraft. The level of vibration and shock depends on the launch vehicle and in the way a CubeSat dispenser (e.g., P-POD) is mounted on the rocket. To qualify for a spaceflight, a CubeSat must undergo vibration testing at the vibration load prescribed for the intended rocket. Such a vibration-certification test occurs after the CubeSat is completely built.

Another area that is excluded from consideration is the effect of space radiation, which is an important consideration for CubeSats flown on long-mission durations or to destinations beyond Earth. NASA’s pair of 6-U CubeSats called Mars Cube One (MarCO-A and MarCO-B) is an example of CubeSats that must be equipped with radiation-tolerant hardware. For most education-class CubeSats, however, space radiation is not a primary concern for several reasons. Education-class CubeSats would fly on a low Earth orbit (LEO) where Earth’s body cuts half of the radiation bombardments. The planet’s magnetosphere provides further protection against space radiation. Perhaps, the most significant factor is that the mission durations of CubeSats are relatively short. For a CubeSat weighing only a few kilograms and flying at the velocity of ∼ 7.8 km/s (17,000 miles per hour), drag due to a trace of atmosphere in LEO would decelerate the spacecraft considerably. The reduction in the flight speeds in turn causes reduction in the altitudes, subjecting the spacecraft further into thicker layers of the atmosphere. Because most CubeSats would burn into the atmosphere within weeks or months at the most, the risk of failure due to cumulative space radiation is relatively low.

### 1.6 Related projects based on Raspberry Pi

As for prototyping of CubeSats, another compelling open hardware is Paspberry Pi, which may be used instead of or in addition to Arduino. One example is the AMSAT CubeSat Simulator (or CubeSat Sim), a Raspberry Pi-based, functional model of CubeSat developed for educational purpose [28]. The CubeSat Simulator can transmit current, voltage, and temperature telemetry on the UHF ham radio band, and re-charge its power using solar panels.

Raspberry Pi is also applied to some balloon projects. In one balloon experiment, involving a five-hour flight at an altitude of 37 km, Raspberry Pi on-board computer was used for CeBr_3_ detector (as well as for the temperature and barometric sensors), which are intended for a 2-U CubeSat [29]. The McGill High-Altitude Balloon (McHAB) team utilized Raspberry Pi processor to control reaction wheel actuators of the balloon payload [30]. Project LEDSAT tested LEO CubeSat on a weather balloon with Raspberry Pi processor and various sensors (i.e., real-time clock module, magnetometer, gyroscope, accelerometer, and GPS receiver) [26]; the goal of LEDSAT was to visually track a CubeSat prototype with light emitting diodes (LEDs) flown at night [26]. Finally, a balloon experiment was conducted for a lightweight, low-cost attitude sensor that runs on Raspberry Pi [31].

## 2 Hardware description

As for Arduino-based CubeSats, demonstrations on prototypes—preliminary mock-up models used as proofs of concepts—have been conducted in the lab settings [5], [6], [7] and on weather balloons at high altitudes. It shall be noted that in this paper the term “prototype” is used broadly, referring to a range of preliminary models that are partially built with various levels of fidelity. The values of balloon demonstrations for such spacecraft prototypes are discussed in *1.3. Background on high altitude testing for student-built prototypes of small satellites*. Davis et al. [32] presented a balloon experiment of student-built, CubeSat form-factor payload based on Arduino or Raspberry Pi which measured the temperature and acceleration during the flight. Among examples unrelated to space applications, Arduino-based instruments were flown on balloons to measure the intensity of the ultraviolet (UV) light and the balloon's position and velocity using an integrated GPS unit [33], as well as radiation and magnetic fields [34]. Despite the advantages of Arduino, the reliability and the accuracy of Arduino-based CubeSats under flight conditions, on balloons or in orbit, are not well examined. One aim of this paper is to contribute to the discussions on this topic.

Although only a few examples of satellites employed APRS in the past, the fact that APRS could rely on an existing network of users is an advantage for education-class CubeSats. Our experiment was indeed carried out using only a flight station (a radio transmitter on the flight payload) without a ground station of our own. Our balloon experiment also serves as testing of an open source, low-cost approach for developing miniature satellites.

As for the choices of sample measurements, tracking the atmospheric temperatures and pressures might appear uncommon as a choice of technology demonstration for CubeSats, which operates mostly outside of the atmosphere. But CubeSats released in orbit will eventually reenter the atmosphere, and spend some time in the top (thin) portions of the atmosphere near the end of their missions. Somewhat surprisingly, properties of the atmosphere at high altitudes are not well understood. The atmospheric properties depend on such factors as locations, time, season, and even sunspot activities. The large uncertainties in the properties of the atmosphere at high altitudes make it difficult to predict the duration of CubeSat missions as well. As such, analysis of atmospheric properties at very high altitudes is a possible study that might be carried out by low-cost CubeSat missions. In this paper the term “prototype” refers to preliminary mock-up, which does not simulate the full capability of CubeSats, but the procedures and methods being described in this paper can be generalized to flight tests of such an open source hardware as Arduino board and its sensors.

Notable features and applications of our hardware and its flight demonstrations include:

• Arduino board and environmental sensors, tested in a space-like environment of high altitudes flown by a weather balloon.
• Thermal and barometric measurements, used for preliminary validation of the accuracy and feasibility.
• Balloon demonstration as a validation test of COTS products for building education-class CubeSats.

## 3 Design files summary

Table 1 is a list of online file repository that was set up for this article. The CAD file (*CubeSat_Prototype_Body_V1.stl*) was used to 3D print the structure of “CubeSat prototype” body being used in the balloon experiment. The Arduino script for thermal and barometric measurements is available as *Arduino_Main.ino*. The two “header files” (used in C/C++) to connect the BME280 and DHT22 environmental sensors to Arduino are *Seeed_BME280.h* and *DHT.h*, respectively. Table 1 does not include information on BigRedBee’s BeeLine APRS transmitter, because no modification was made to this COTS product.

**Table 1:** Design files for our the flight payload.

Design file name	File type	Open Source License	Location of the File
CubeSat_Prototype_Body_V1.stl	CAD	GNU GPL 3.0	Article’s repository
Arduino_Main.ino	Software	GNU GPL 3.0	Article’s repository
Seeed_BME280.h	Software	MIT License	https://github.com/Seeed-Studio/Grove_BME280
DHT.h	Software	MIT License	https://www.arduino.cc/reference/en/libraries/dht-sensor-library/

## 4 Bill of materials summary

Table 2 shows electronic components used in our flight payload (“CubeSat prototype”), with ∼$374 being the total cost. This bill of materials does not include the auxiliary items used to carry out the balloon flight. The primary auxiliary items include the camera to record the video footage of the payload during the flight, the secondary tracking system (SPOT GPS) and its subscription fee, the weather balloon and parachute, and the helium tank.

**Table 2:** Bill of materials for the flight payload.

Designator	Component	Number	Cost per unit, USD	Total cost, USD	Source of materials	Material type
Arduino	Arduino Uno R3 Board	1	$23	$23	https://store-usa.arduino.cc/products/arduino-uno-rev3/	Composite
Breadboard	Breadboard	1	$5	$5	https://www.adafruit.com/product/65	Composite
DHT	DHT22	1	$10	$10	https://www.adafruit.com/product/385	Composite
BME	BME280	1	$22	$22	https://www.diymalls.com/DIYmall-GY-BMEP-5V-BME280-Pressure-Humidity-Temperature-Sensor?search=bme%20280	Composite
microSD card module	MicroSD card breakout board+	1	$8	$8	https://www.adafruit.com/product/254	Composite
microSD card	MicroSD card	1	$6	$6	https://www.westerndigital.com/products/memory-cards/sandisk-ultra-uhs-i-microsd#SDSQUNC-016G-AN6MA	Composite
APRS	BigRedBee 2-Meter 5-Watt APRS Transmitter	1	$265	$265	https://shop.bigredbee.com/products/2-meter-5-watt-aprs-transmitter	Composite
Antenna	BigRedBee VHF Dipole Antenna	1	$35	$35	https://shop.bigredbee.com/products/brb-vh-dipole-antenna	Metal
CubeSat Prototype Body	3D-printed structure (PLA filament)	1	Supply	N/A	https://www.hatchbox3d.com/collections/pla-1-75mm	Polymer

## 5 Build instructions

### 5.1 The interior: Arduino

**Fig. 2:**
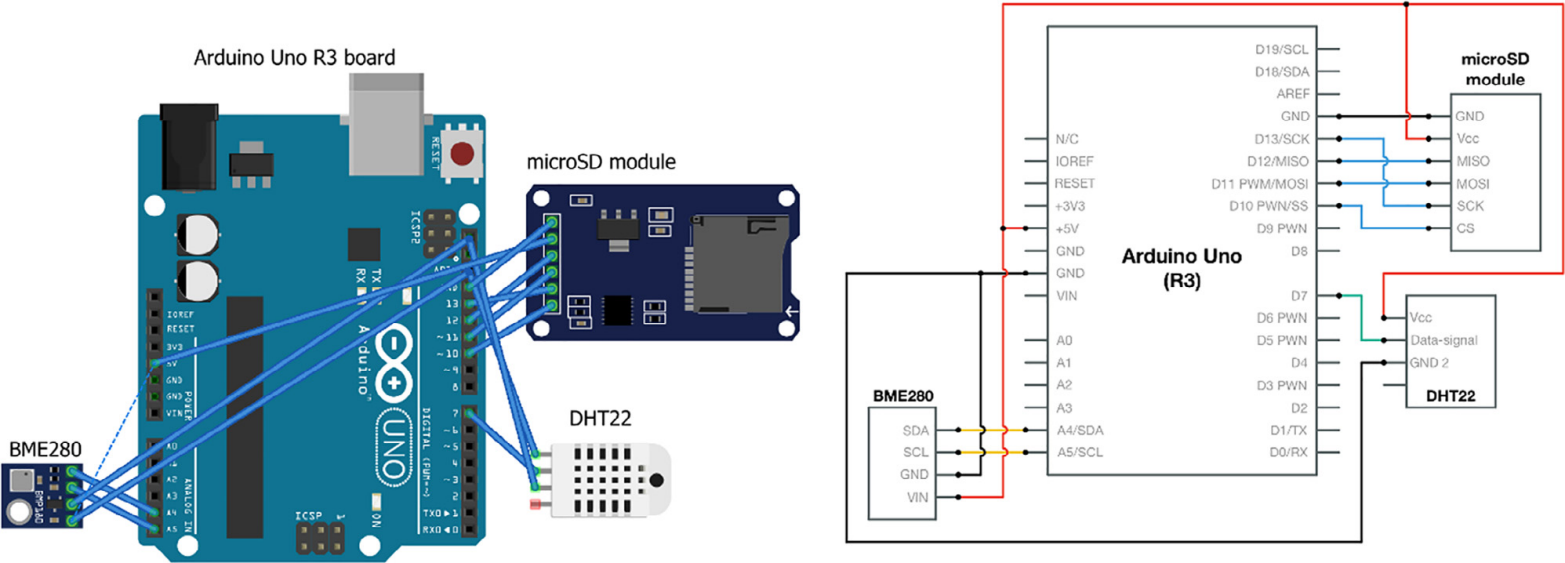
Schematics and circuit diagram of Arduino board.

The schematic drawing of the Arduino board is presented in Fig. 2 (left). The Arduino circuit includes the Arduino Uno R3 board, the BME280 environmental sensor (which can measure the temperature, humidity, and atmospheric pressure), the DHT22 thermal humidity sensor, and the microSD breakout board. The external (i.e., ambient) temperature and pressure were measured using the BME280 sensor, which, according to the manufacturer, has an operational range of −40 °C to 85 °C with an accuracy of ±1 °C. The DHT22 sensor was used to measure the temperature inside the “CubeSat structure,” which was used in a separate study [7].

**Fig. 3:**
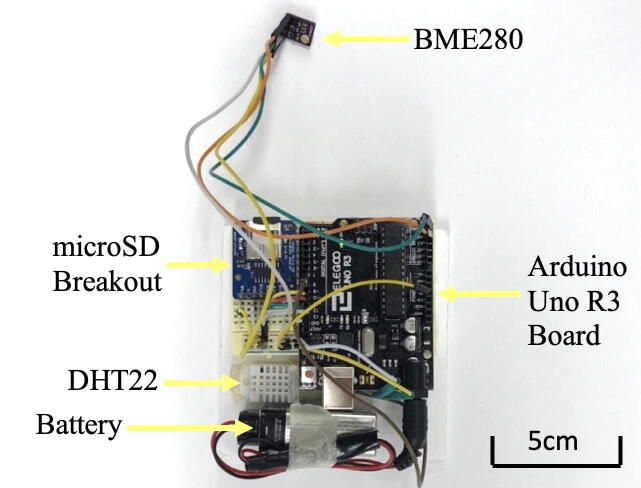
Our flight payload contains the Arduino circuit.

A detailed circuit schematic is shown in Fig. 2 (right). Both sensors and a microSD module are connected to run on 5 V. Apart from the +5 V pin and GND pin, the BME280 sensor is plugged into pin A4 and A5, the DHT22 sensor is connected to the Arduino Uno through pin D7, and the microSD module uses pin D10, D11, D12, and D13. Detailed instructions are provided in the webpages listed in Table 3. The assembled model is shown in Fig. 3.

**Table 3:** Web-based resources on more detailed instructions.

	Topics	Instructions and resources
a	Connecting a DHT sensor to Arduino	https://learn.adafruit.com/dht/connecting-to-a-dhtxx-sensor
b	Connecting a BME sensor to Arduino	https://learn.adafruit.com/adafruit-bme280-humidity-barometric-pressure–temperature-sensor-breakout/arduino-test
c	Connecting a microSD card breakout board to Arduino	https://learn.adafruit.com/adafruit-micro-sd-breakout-board-card-tutorial/arduino-wiring

### 5.2 The interior: APRS

The radio communication makes use of the Automatic Packet Reporting System (APRS). As discussed earlier, the range of radio frequencies used by APRS is 144.39–145.825 MHz (i.e., wavelengths of 2.06–2.07 m), which includes 144.39 MHz for North America (where the balloon test is conducted) and 145.825 MHz for “space.”.

**Fig. 4:**
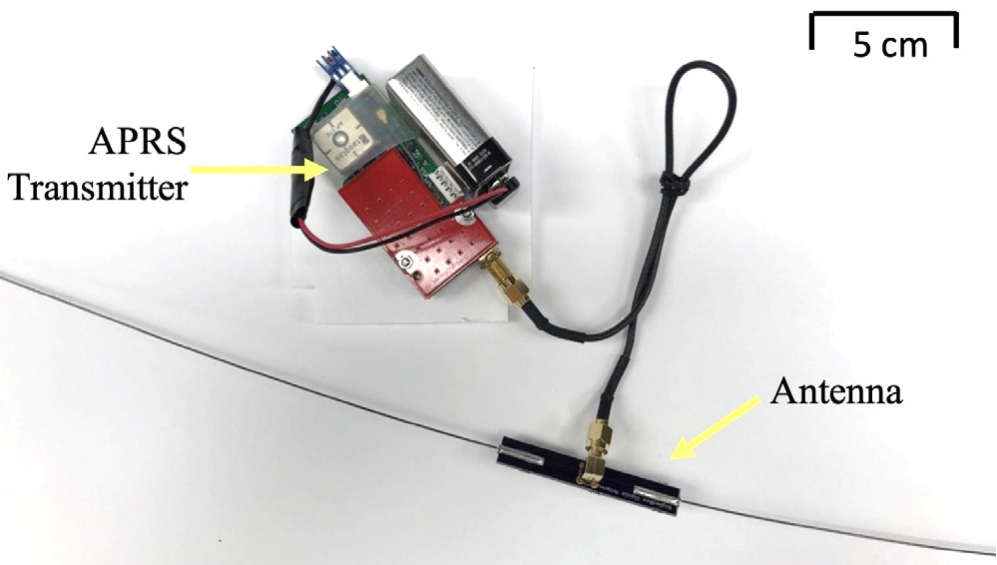
Our CubeSat prototype contains the APRS transmitter.

The particular hardware used in our experiment was a BigRedBee 2-Meter 5-Watt APRS Transmitter (where “2 m” refers to the wavelength). The length of the dipole antenna is 1 m, half of the wavelength. Before the first usage, the APRS/GPS transmitter was configured using BigRedBee’s GPS programming software. The program allows the user’s callsign to be paired with the device. The rate of transmission can also be changed at this stage. Once the antenna and power source (i.e., the 9 V battery) are attached onto the APRS transmitter, the APRS is ready for use (Fig. 4). (The GPS data was used for validation of the altimeter data and for tracking during the balloon test. This COTS GPS unit cannot be used for spaceflight.).

### 5.3 3D printing of CubeSat-inspired flight payload and assembly of the balloon payload

**Fig. 5:**
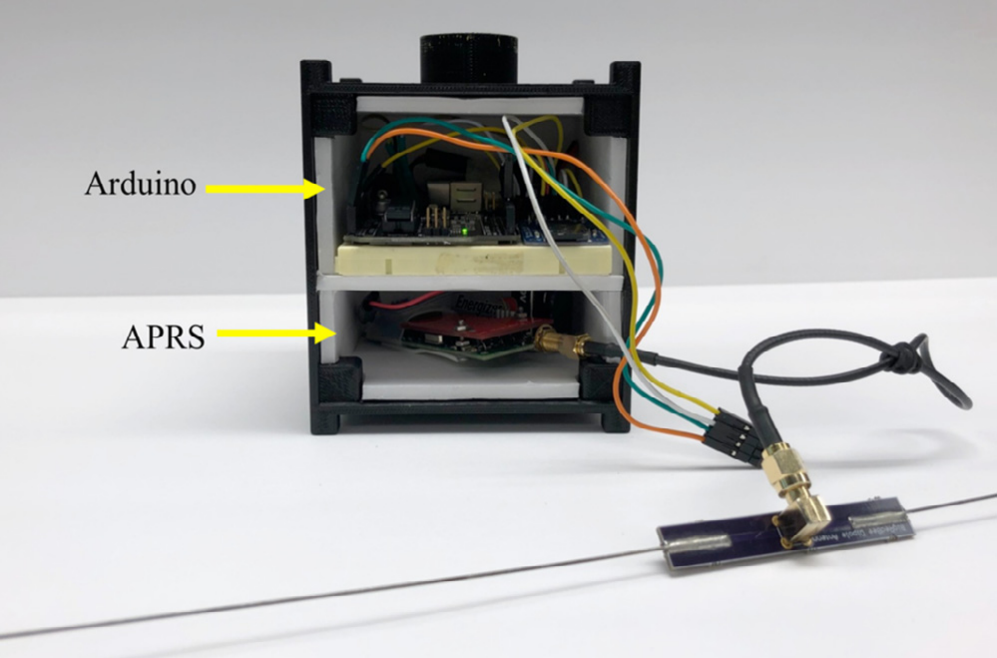
Internal view of the “CubeSat prototype” containing Arduino and APRS modules.

Due to safety concerns, the structure used for the flight demonstration was not made of aluminum typically used for CubeSats, but 3D printed using polylactic acid (PLA). The 3D-printed PLA structure had a wall thickness of 3 mm. Polystyrene foam boards with a thickness of 4 mm were used as an inner layer (Fig. 5) to provide some thermal insulation. The dimension of the 3D printed structure conformed to the standard for 1-U CubeSats (and can be 3D printed using the CAD file *CubeSat_Prototype_Body_V1.stl*). The interior of the 3D printed CubeSat body bifurcates into the top and bottom shelves: the Arduino Board on the top shelf and APRS on the bottom shelf (Fig. 5). There is a cable, which runs through a drilled hole on the access panel (i.e., removable wall), connecting the APRS transmitter placed on the bottom shelf and its antenna mounted outside of the body. The access panel is then screwed onto the CubeSat body to complete the assembly. Prior to the flight experiment, the Arduino and APRS are turned on immediately before closing the access panel.

**Fig. 6:**
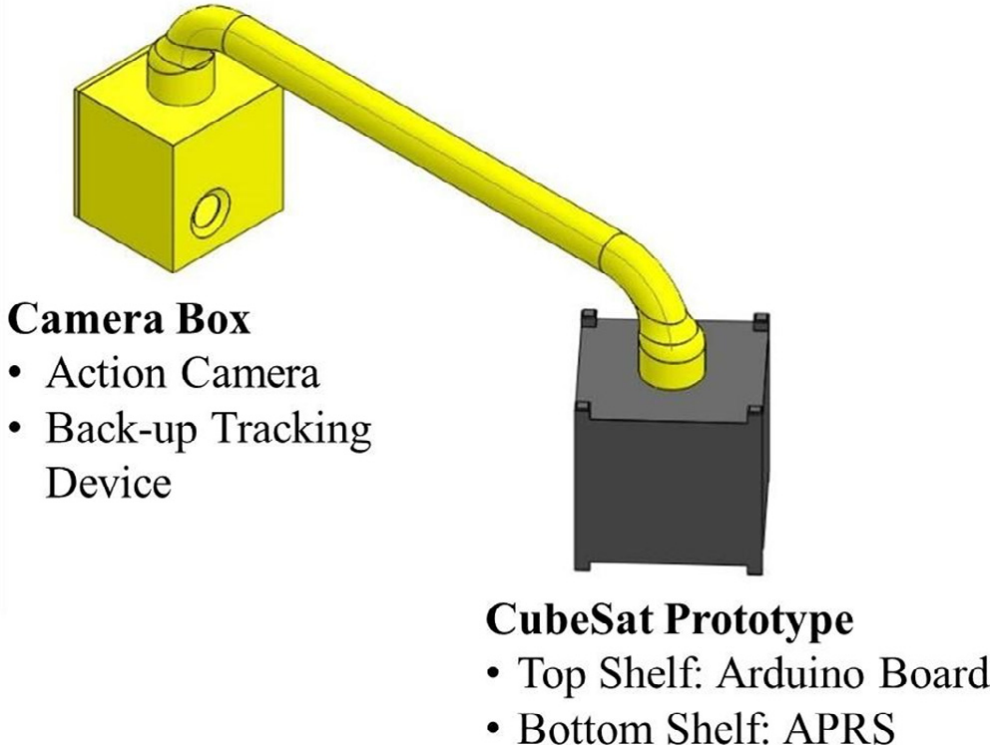
The configuration of our balloon payload.

The balloon payload consists of the “CubeSat prototype” and a separate “camera box” containing a camera and a backup tracking device (i.e., SPOT GPS) (Fig. 6). The 3D printed structural casing created for the balloon experiment has a cylindrical extruded section on top, which connects with the elbow joint of the PVC pipe. The camera is pointed at the prototype body for monitoring.

The CubeSat-inspired mock up used for the balloon flight is made for the purpose of balloon flight to validate Arduino and its sensors; the mock up is not intended to simulate the all features of actual CubeSats. In our mock up, the Arduino circuit was not integrated into APRS (so it transmits the GPS coordinates, but not the temperature or pressure data), the batteries cannot recharge via solar panels, and the radio antenna was simply attached outside of the 10 cm structural frame (rather than being deployed during the flight).

### 5.4 The CNC machining of aluminum structure

Although the prototype structure for the balloon experiment was 3D printed using PLA, an aluminum structure that conforms to the CubeSat standard was also fabricated for the purpose of lab studies. This section provides a brief overview of the fabrication process of the aluminum body.

For CubeSats flown on the actual space missions, the structures must be made with the dimensional accuracy of <0.1 mm using specific types of aluminum alloys, with the most common choices being the 5005, 5052, 6061, or 7075 aluminum [35]. Our structure was made from multiple pieces made of the 7075 T6 aluminum.

**Fig. 7:**
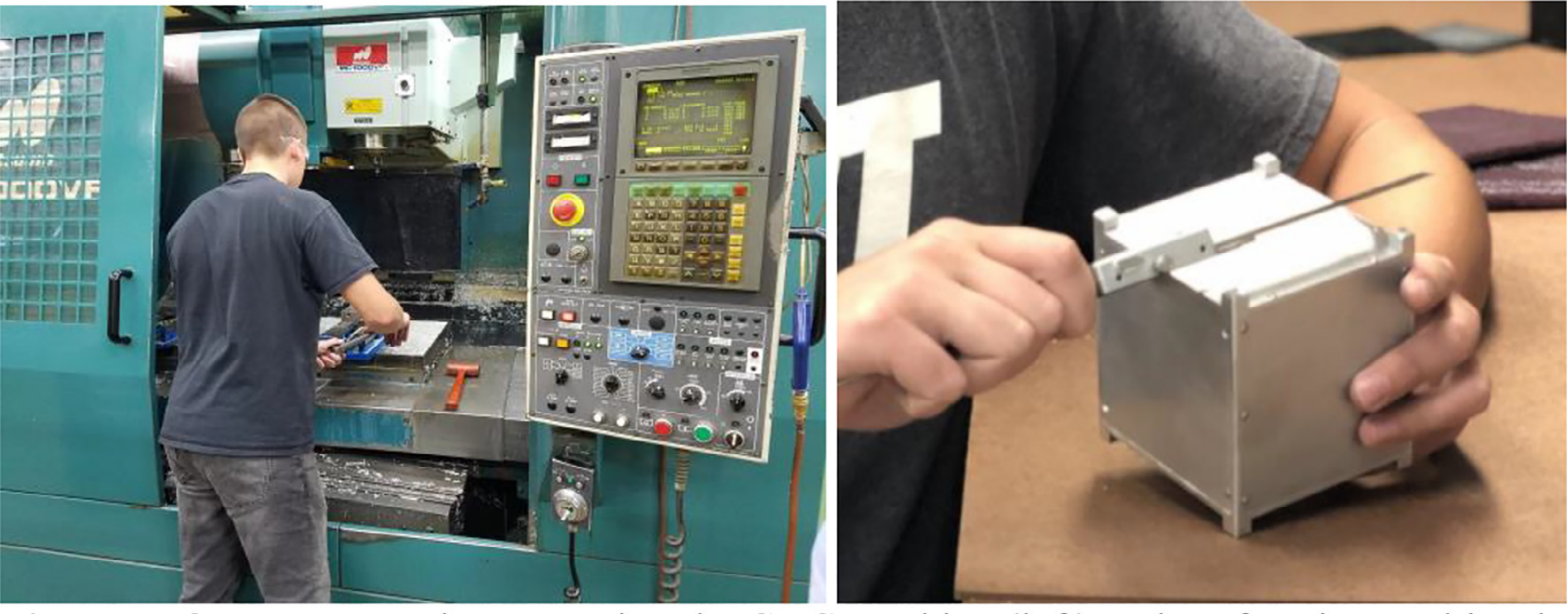
Our team member operating the CNC machine (left) and performing multi-point measurements using a digital caliper (right).

The aluminum pieces were processed using the computer numerical control (CNC) machine (Fig. 7), whereas the Mastercam program was used to design the toolpaths. These tasks were not discrete, sequential steps in practice, as it was often necessary to go back and forth between the CNC machine and the Mastercam program several times to ensure that each piece was fabricated correctly. The CNC-machined aluminum parts were then inspected using the optical comparator, which allows precise measurements of parts from the magnified shadows casted on a glass plate. Minor adjustments were made by shaving off aluminum further, a process that had to be done carefully as shaved aluminum could not be put back. The overall flatness of each part was ensured using the dial indicator on a granite surface plate. To create the mirror finishing appearance, the surface was treated using fine glass beads and polished using scouring pads. Once each of the aluminum parts was fabricated accurately, they were assembled using screws.

Multi-point measurements using a digital caliper confirmed that the assembled structure satisfied the CubeSat standards, within the allowable error of <0.1 mm from the nominal values. In fact, the maximum deviations observed in lengths, widths, and depths was 0.05 mm. The chamfer was within 0.05 mm (which CubeSat standard also requires).

Although an undergraduate student carried out the entire tasks described above, the process required close supervision and frequent guidance from a trained machinist who also provided assistance for such tasks as writing a program and selecting the most efficient tools. The labor by the student member was ∼45 h (including planning, ∼18 h of machining, final assembly, and inspections), but substantially longer time would have taken if the volunteer guidance from the professional machinist were unavailable.

### 5.5 Future work

In this paper the term “prototype” is used for our mock-up model, which was not intended to simulate all features of spacecraft. Most CubeSats have a computer-on-a-chip (which could potentially be Arduino), sensors, a radio transceiver, and a power system. Our next step is to interface Arduino with the transceiver, so that collected telemetry and housekeeping data can be transmitted to the ground station in real time (rather than being stored locally). In addition to downlink, it is also desirable to have an uplink for commanding the spacecraft. There are advantages in validating hardware through various tests (including balloon tests) before undertaking software development for the chosen set of hardware. The quality of scientific investigation performed by CubeSats would improve with the quantity of the data gathered. Extending the duration of data gathering—from hours to months—in turn means that batteries need to be recharged via solar panels. Therefore, building the power system (including rechargeable batteries, solar cells, and power bus) and associated software is another area of future work.

## 6 Operation instructions

Prior to the balloon launch, only a few steps were required to start Arduino and APRS. The assembled Arduino circuit can be turned on by simply plugging the power source into it. Blinking on the microSD module on the Arduino board indicates that the data recording has started. The blinking rate is identical to the data sampling rate, which is set to one measurement every 15 s in our case. Operation of APRS can be started by connecting the APRS transmitter to the power source (9 V battery). The GPS unit would automatically start searching for the GPS satellite signals. Once engaged, the APRS begins transmitting its position.

## 7 Validation and characterization

The atmospheric pressures measured by Arduino’s environmental sensor were converted into the pressure altitudes, so they can be compared against the GPS altitudes transmitted via APRS. The sensor software library comes with a built-in function to convert the measured pressure to the estimated altitudes using the standard atmosphere model [36]:

(1) $$h_{p}=\frac{T_{o}}{\beta }\left[1-{\left(\frac{P}{P_{o}}\right)}^{0.1903}\right]$$

where *h*_p_ is the pressure altitude, *P* is the measured pressure, *P*_o_ is the standard day pressure at the sea level, *T*_o_ is the standard day temperature at the sea level, and *β* is the temperature lapse rate with respect to the altitudes. Using the data from the International Organization for Standardization [20], the values of the constants used in Eq. (1) are *P*_o_ = 101,325 Pa, *T*_o_ = 288.15 K, *β* =  −6.5℃/km for altitudes below 11 km. The equation is invalid for altitudes above 11 km. More discussions on Eq. (1) are found in Ref. [37].

It shall be noted that comparing the Arduino measurements against the GPS altitudes can be used for preliminary validation only. The two data sets are not expected to be exactly identical for several reasons. First, the two altitudes were actually measured from different reference points: the pressure altitude’s reference is the mean sea level (in which the sea level conditions are assumed to be 101,325 Pa and 288.15 K), whereas the GPS altitude’s reference is the ellipsoid that approximates the surface of the Earth. The difference in these two measurements can be tens of meters. Second, the atmospheric condition deviates, often significantly, from the standard atmosphere depending on the time and locations. Those deviations are readily observed in the examples by Sobester [38]. Third, Eq. (1) is valid only up to the altitude of 11 km. Fourth, the sparseness of measurements and transmissions translates into additional uncertainties in the estimated altitudes. In our balloon flight, for example, the rates of ascent and descent are approximately 4 m/s and 8 m/s, respectively. So, during the 15 s of interval used by Arduino to measure atmospheric pressures, the altitudes would have changed 60 m and 120 m, respectively. Finally, the GPS altitudes in general—not just those sent via APRS—can have errors of tens of meters in the vertical positions (even though errors in the horizontal positions are much smaller).

**Fig. 8:**
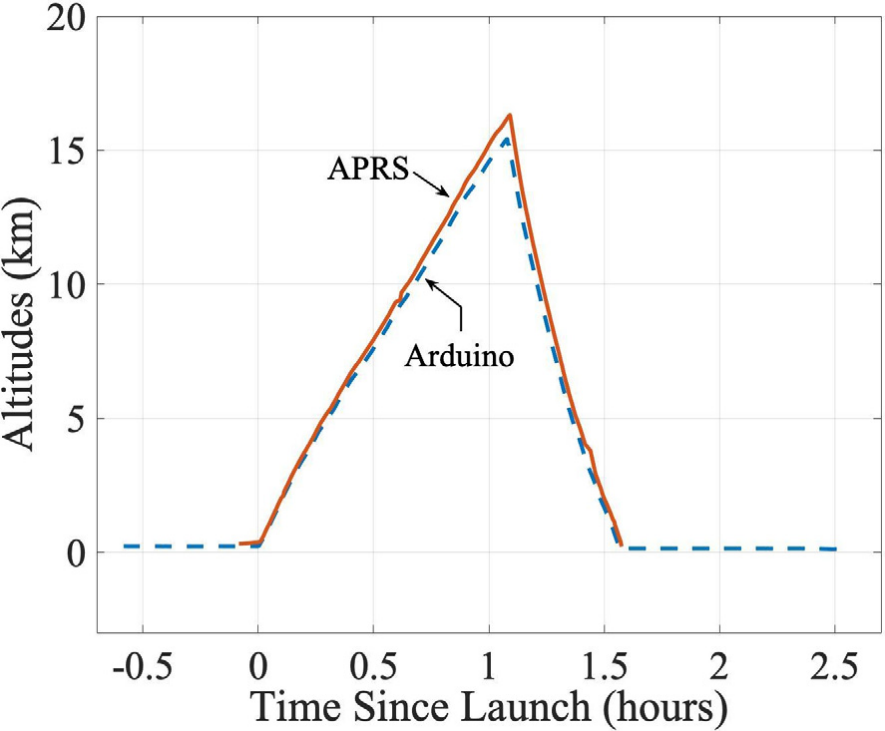
Altitude as reported by Arduino and APRS.

Fig. 8 shows the pressure altitudes measured by Arduino and the GPS altitude transmitted by APRS. The horizontal axis shows the time since the moment of launch (at 9:45 AM of the flight day). As expected, the two sets of data follow close to each other (except at altitudes >11 km when the equation used to calculate the pressure altitudes is invalid).

The “altitudes” reported by APRS and Arduino for the launch site, the highest altitude, and the landing site are compared in Table 4. As is often the case for a hot summer day, our pressure altitudes are reported lower than the GPS altitudes. At the launch and the landing sites, the Arduino estimates on the elevations were lower by 88 m and 71 m, respectively, than those reported by GPS. The difference between the APRS and the Arduino altitudes expands to 897 m by the time the balloon reaches the highest altitude (16 km). The larger mismatch at the high altitudes can be explained by the modeling error in Eq. (1) above 11 km and the model itself assumes a standard atmosphere. In this sample experiment, therefore, the measurements using the BME280 environmental sensor integrated into our Arduino-based measurements were validated within the expected errors.

**Table 4:** **“**Altitudes” reported by APRS and Arduino.

	Launch site a [m]	Highest altitude [m]	Landing site b [m]
GPS altitude from APRS	306.93	16,315	210.92
Pressure altitude from Arduino	219.14	15,418	140.1

a Greenwood Furnace State Park, Huntingdon, Pennsylvania (latitude 40.651°, longitude −77.757°).

b East of Mount Pleasant Mills, Pennsylvania (latitude 40.697°, longitude −76.957°).

**Fig. 9:**
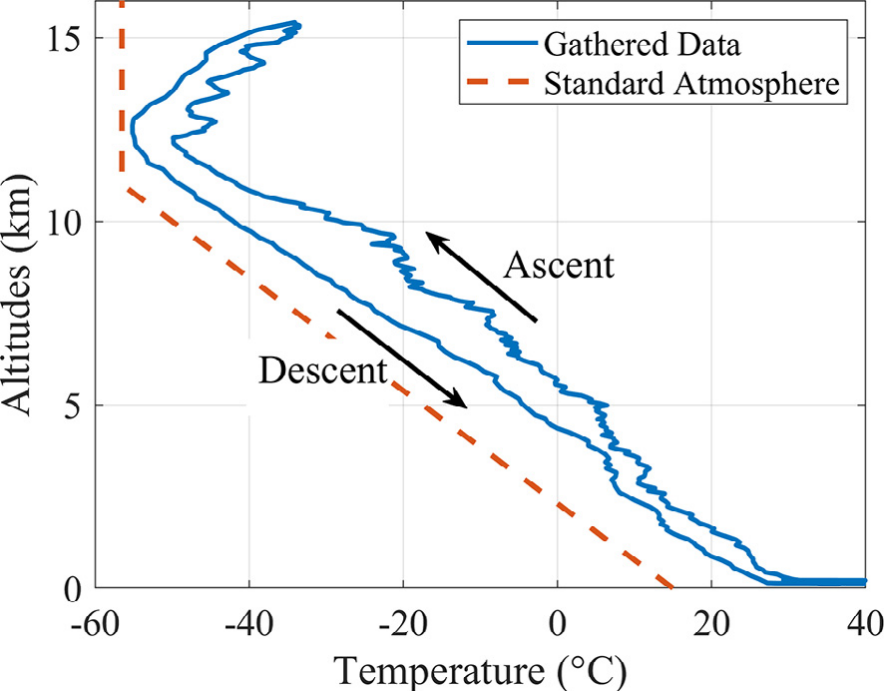
Temperature measured by Arduino’s thermal sensor.

The BMP280 sensor we used to measure atmospheric pressure is also capable of measuring temperature (Fig. 9), and is included here as supplemental data. The lowest recorded temperature by the sensor was −55.1 °C, which is close to the expected temperature of –56.5 °C for altitudes 11–20 km, according to the Standard Atmosphere model. This balloon experiment took place in summer; the measured temperatures were higher than what Standard Atmosphere would predict for the corresponding altitudes.

The lower bound of the operating temperature for DHT22, BME280, Arduino Uno, and lithium ion battery are all listed as –40 °C, according to the respective manufacturers. But, as discussed earlier, the DHT22 sensor stopped its measurement exactly at −40 °C whereas the BME280 continued to operate at −60 °C (although the accuracy below the operational limit was not validated). Arduino Uno could potentially operate at even lower temperature (still unconfirmed), but the battery that powers it cannot be subject to low temperature for too long. These test results suggest that the BME280 sensor can be placed outside the prototype body, but the DHT22 sensor and the battery must be housed inside an enclosed casing with some thermal insulation, as demonstrated in our balloon flight.

## 8 Conclusion

Education-class CubeSats are characterized by a heavy reliance on commercial off-the-shelf (COTS) products, which helps reduce the cost of the project. But there is a familiar trade-off: using parts that are not designed for the intended space missions also means low performance, low accuracy, and low reliability. These limitations can be mitigated by verification tests on the selected parts or on the assembled spacecraft. While there are effective preliminary lab tests that can be performed relatively easily and inexpensively, no lab equipment could test the integrated CubeSat system, including long-range communications. Balloon flights can be used to subject the hardware to harsh space-like environments over ranges of altitudes and distances.

Arduino is a promising flight computer for entry-level CubeSats, yet the literature on Arduino-based CubeSats (or even on Arduino-based prototype studies of CubeSats) is limited. This paper contributes to this discussion by presenting a hardware verification by flying a mock-up model on a weather balloon. The Arduino sensor measured the atmospheric pressures for the entire range of the altitudes flown, and the calculated pressure altitudes showed sufficient agreement with the transmitted GPS altitudes.

## CRediT authorship contribution statement

**Kenjiro Lay**: Data curation, Investigation, Methodology, Software, Validation, Visualization, Writing - original draft, Writing - review & editing. **Lingqi Li**: Investigation, Software, Writing - original draft, Writing - review & editing. **Masataka Okutsu**: Conceptualization, Funding acquisition, Project administration, Supervision, Validation, Writing - original draft, Writing - review & editing.

## Declaration of competing interest

The authors declare that they have no known competing financial interests or personal relationships that could have appeared to influence the work reported in this paper.

## Figures and Tables

**Fig. A1:**
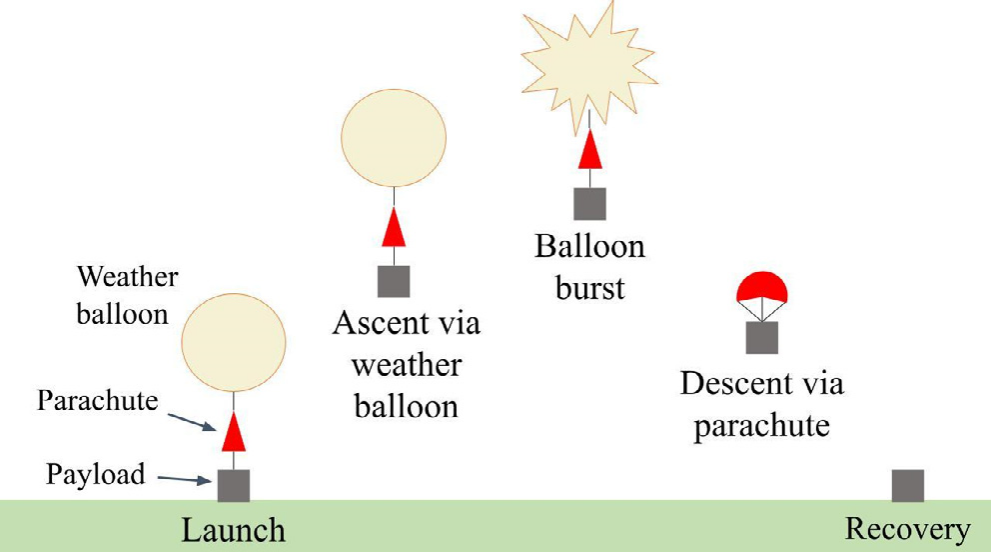
Flight phases of a weather balloon.

**Fig. A2:**
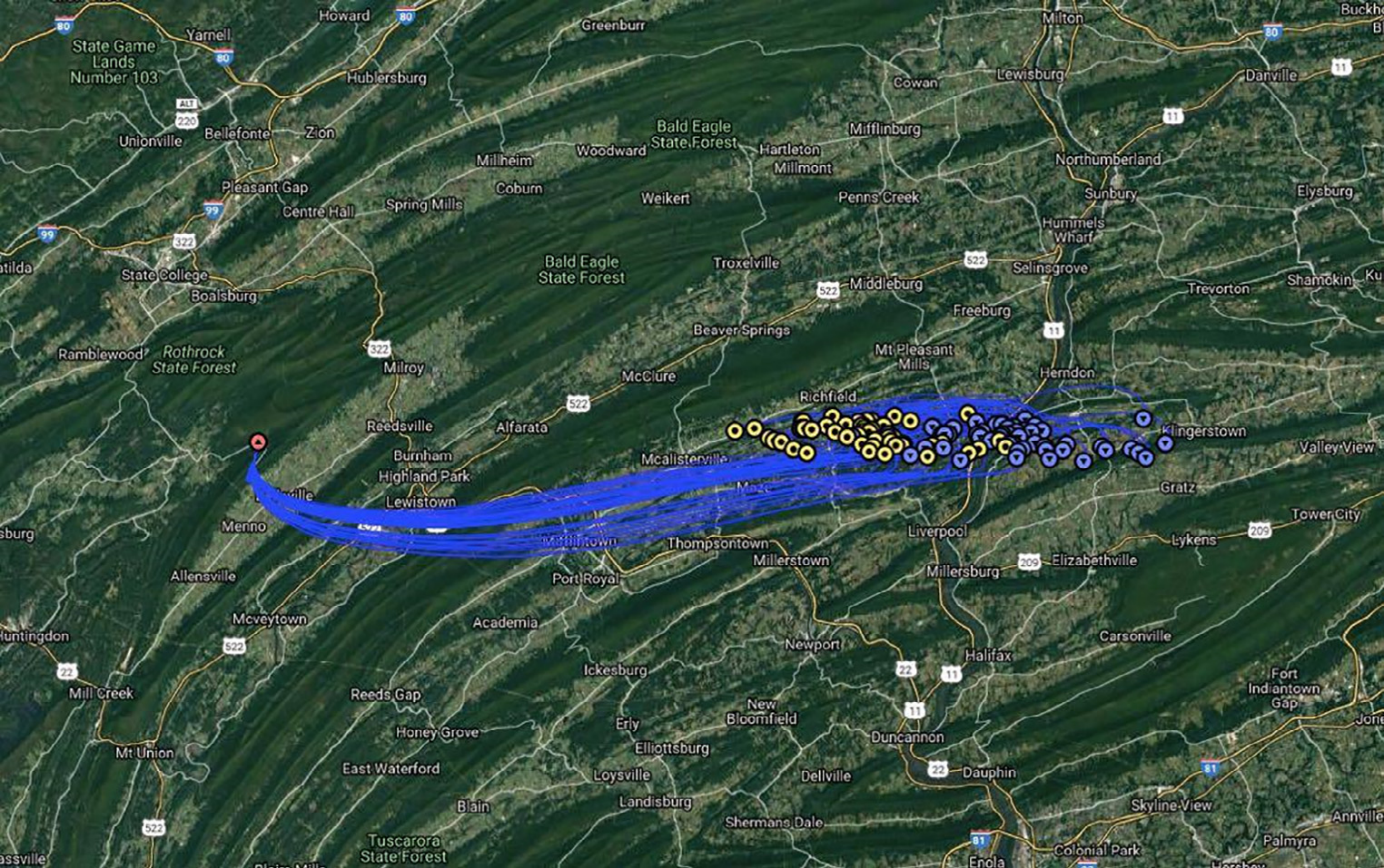
Typical flight simulation results from ASTRA High Altitude Balloon Flight Planner.

**Fig. A3:**
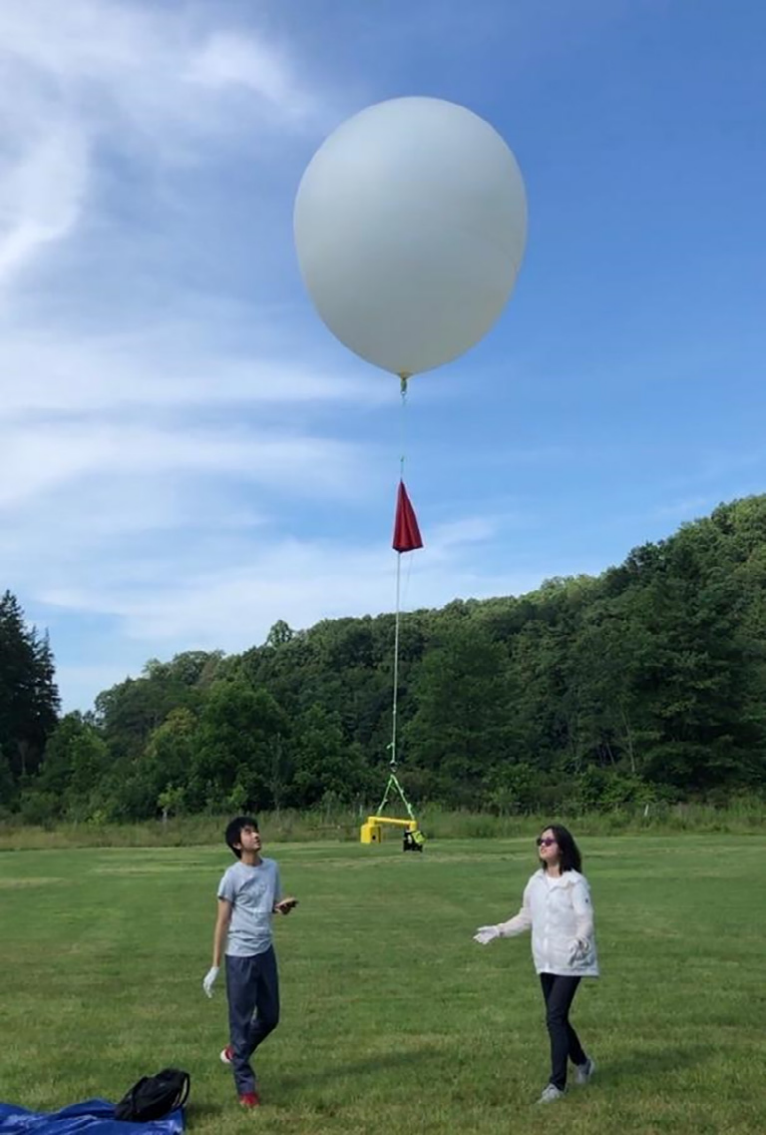
Our CubeSat-inpired payload, tethered to the weather balloon, was released from a state park located in central Pennsylvania.

**Fig. A4:**
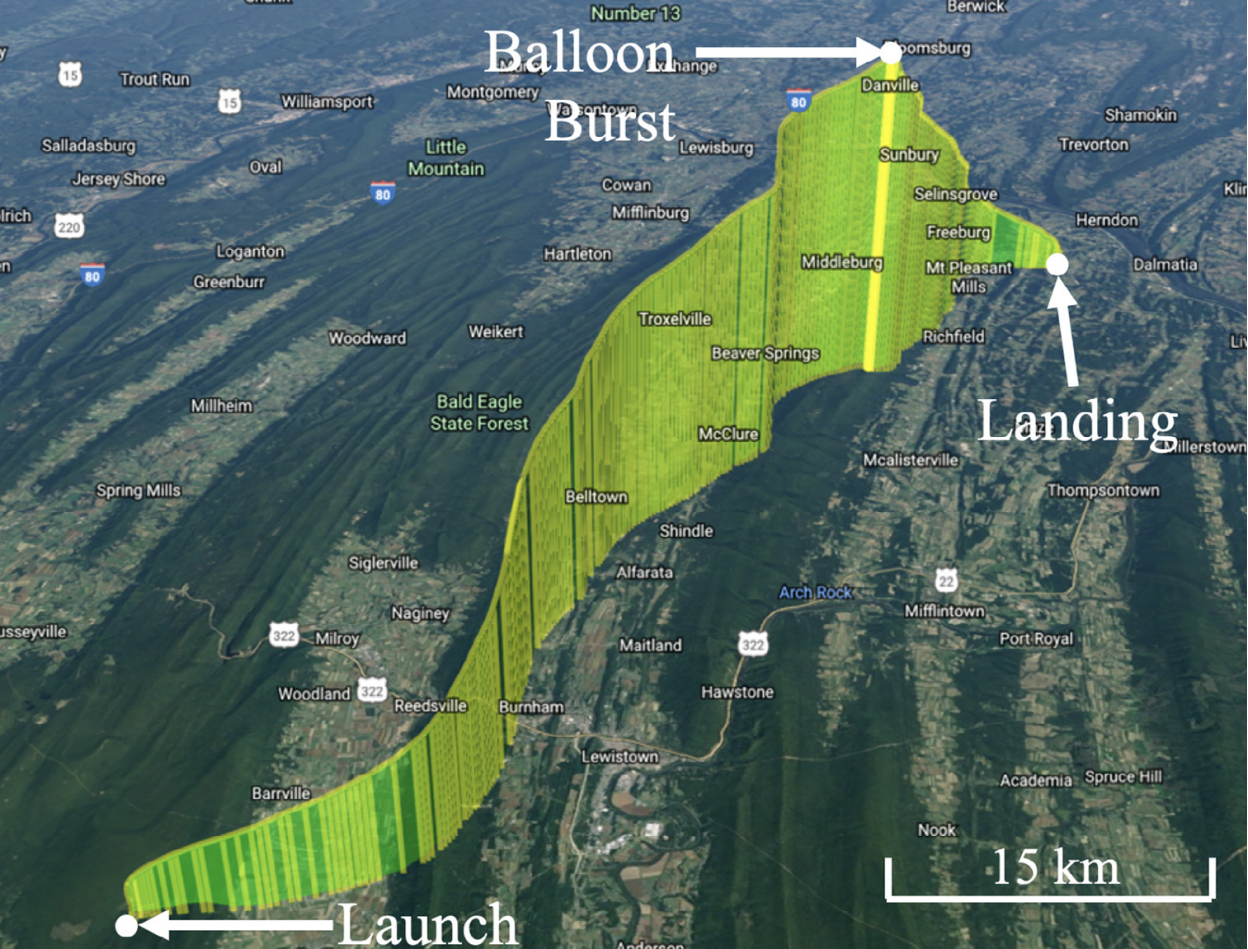
3D modeled tracking results produced by APRS, the balloon landed approximately 70 km east of the launch site.

**Fig. A5:**
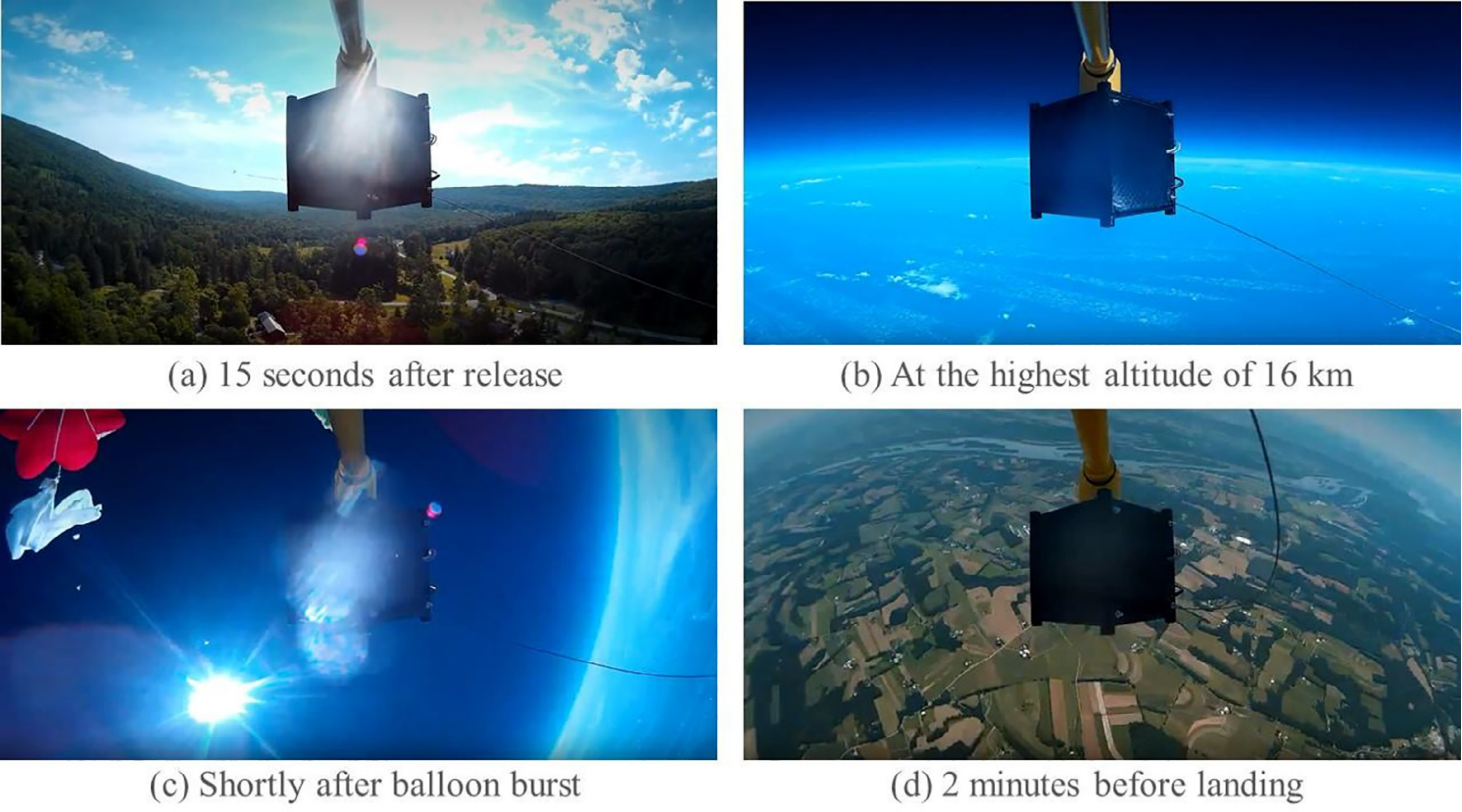
Screen captures of the recordings from the balloon flight. The apparent curvature of horizons is caused by the camera’s wide-angle lens.

## Appendix

There are logistics involved in arranging high-altitude testing. For the interested readers, this Appendix provides planning and procedure of the experiment.

### A.1 Flight planning

When conducting balloon flights (Fig. A.1), safety is the top priority. Much of our activities took place at our university’s Abington campus, located in the northern suburb of Philadelphia, but the balloon could not be released from the campus, because there were two nearby airports (PHL and PNE) and the flight downrange included populated areas between Philadelphia and New York. After considering all logistical factors, a state park in central Pennsylvania was chosen as the launch site, and the permission from the park was obtained.

The drawback of this state park was that it did not have cellular or wifi coverages. At the moment of launch, the team members’ laptop and smartphone screens could not retrieve the GPS coordinates transmitted via APRS. The balloon had to be released “blind.” Another challenge of the chosen launch site was that it was located deep within the mountainous forest, where the landed payload could get caught on a high branch of a tree. Forests make it difficult to find and recover the payload, even if permission to enter those areas could be obtained.

Thus, the balloon had to fly far enough to clear the mountainous areas to reach open fields, but not too far to reach the populated areas. The balloon’s flight path also depended on the weather pattern of the launch date. The weather could not be too windy, rainy, foggy, or cloudy (as set forth in CFR Title 14 Part 101). During the window of launch dates, the team was on standby, monitoring the weather conditions daily to ensure that conditions are favorable.

### A.2 Trajectory simulation

The prediction of the landing location was enabled using the ASTRA High Altitude Balloon Flight Planner [39], [40], a free web-based trajectory simulation tool. The input to the simulator includes the launch conditions (i.e., coordinates, date, and time) and the balloon parameters (i.e., the balloon dimension, payload weight, and positive lift). The required amount of helium, which was one of the input parameters for the ASTRA program, was estimated using the Balloon Performance Calculator [41].

The ASTRA program performs the Monte Carlo simulation for probabilistic distributions of the trajectories [39]. Fig. A.2 is one of our simulation results in which 50 trajectories were propagated from the intended launch site (marked with a red dot). Once launched, the balloon would drift along with the wind, blowing generally eastward. At high altitudes, wind direction could sometimes reverse, an effect observed also in Fig. A.2. The yellow dots are where the balloon is predicted to burst, marking the highest altitudes reached by the balloon. Once the balloon bursts, the payload descends via parachute. The landing locations are marked with blue dots. Compared to the ascent speed, the descent speed is greater, so the horizontal drifts during the descent are shorter than during the ascent. When planning for flights, we would zoom into the map to examine the entire landing zone for potential concerns. The targeted landing areas were open fields, being away from the mountains and populated areas.

### A.3 Regulatory issues

When conducting weather balloon flights in the United States, there are regulatory issues concerning two government agencies: Federal Communication Commission (FCC) and Federal Aviation Administration (FAA).

Operation of an amateur radio must comply with FCC per Code of Federal Regulation (CFR) Title 47 Part 97. Student members obtained amateur radio licenses issued by FCC. Our balloon experiment was conducted under the first author’s callsign (KC3LOZ). The flight of the balloon must comply with the FAA regulations. According to CFR Title 14 Part 101, small balloons carrying an individual payload less than 4 lb (1.81 kg) and the total payload less than 12 lb (5.44 kg) (depending on the density) are exempt from adding such safety measures as the payload cut down system, termination system, and radar reflector. Prior to the flight, the team would contact the FAA to file a notice to airmen (NOTAM), which alerts the pilots in the vicinity of potential hazards along their flight path. (Information to be included in NOTAM is launch coordinates, altitudes and direction of flight, and launch and landing time. NOTAM can be filed between 4 and 24 h before flight and can be amended after filing, if needed.).

### A.4 Launch, tracking, and recovery

Our balloon flight took place on June 27, 2019. After arriving at the launch site, the team carried out the procedure they had already practiced on the campus: connect Arduino and APRS to the battery, turn on the camera and the backup GPS device, and inflate the balloon using the helium tank. The balloon was released at 9:45 AM (Fig. A.3).

As soon as the released balloon ascended into the cloud, the team packed the gears and started driving. The GPS coordinates sent by APRS are made available on the APRS’s webpage (aprs.fi), but due to the lack of cellular and wifi coverage at the launch site, the location of the balloon was not immediately known. From the preflight simulations, the team knew that they needed to drive eastward. After driving the mountain road for approximately 15 min, they reached the area with cellular coverage, and the balloon’s location started appearing on the map on the smartphone. With real-time tracking, the team chased the balloon flying many miles above the ground.

After half an hour of driving, the altitudes sent by APRS began to drop. The balloon burst at an altitude of 16.3 km. The balloon payload landed at 11:20 AM in an open field, approximately 70 km from the launch site. The resulting flight duration was 1 h and 35 min. With the position information sent via APRS, the team had no difficulty finding the landed payload in the field. The balloon payload was recovered at 12:06 PM.

Fig. A.4 shows the trajectory of our balloon, reconstructed from the GPS data received on that day. The APRS was set to transmit its GPS coordinates every 15 s. The position data sent via APRS may have errors on the order of several meters (based on our experiences of using it on the ground), but such an error is negligibly small in a plot of trajectory spanning tens of kilometers. Also from our experiences, we were aware that some of the data sent via APRS may be corrupted or not even received. (It is obvious when the data were corrupted, as the indicated locations may not even be in the same continent. The corrupted position data—estimated to be 5–10% of data received during the flight—were manually removed from Fig. A.4.).

The balloon payload included a video camera pointed at the prototype body (Fig. A.5). If, for example, APRS stopped working due to the damage to the antenna by a rope (which could occur during tumbling upon balloon burst), we would be able to find out from the video footage.

Fig. A.5 shows screen captures from the video footage taken during the flight. As discussed earlier, the balloon was released from an area surrounded by a mountainous forest (Fig. A.5a). The highest altitude was 16.3 km (i.e., 53 thousand feet) (Fig. A.5b). A rough tumble is observed at the moment of the balloon burst (Fig. A.5c), which shows the white balloon and the red parachute. After the burst of the balloon, the payload descended via parachute. Fig. A.5d is a video capture at 2 min before landing. The targeted open fields are seen in the view.
